# Transglutaminase 2 Depletion Attenuates α-Synuclein Mediated Toxicity in Mice

**DOI:** 10.1016/j.neuroscience.2020.05.047

**Published:** 2020-06-02

**Authors:** Jie Zhang, Hilary Grosso Jasutkar, Run Yan, Jong-Min Woo, Kang-Woo Lee, Joo-Young Im, Eunsung Junn, Siiri E. Iismaa, M. Maral Mouradian

**Affiliations:** a Robert Wood Johnson Medical School Institute for Neurological Therapeutics, and Department of Neurology, Rutgers Biomedical and Health Sciences, Piscataway, NJ 08854, USA; b Victor Chang Cardiac Research Institute, Darlinghurst, 2010, NSW, Australia; c University of NSW, Kensington 2052, NSW, Australia

**Keywords:** TG2, α-synuclein, protein aggregation, neuroinflammation, Parkinson disease

## Abstract

α-Synuclein (α-Syn) is a key pathogenic protein in α-synucleinopathies including Parkinson disease (PD) and Dementia with Lewy Bodies. The aggregation of α-Syn is believed to be deleterious and a critical step leading to neuronal dysfunction and death. One of the factors that may contribute to the initial steps of this aggregation is crosslinking through transglutaminase 2 (TG2). We previously demonstrated that overexpression of TG2 exacerbates α-Syn toxicity in mice and yeast by increasing the higher-order species of α-Syn. Herein, we investigated whether deletion of the TG2 encoding gene could mitigate the toxicity of α-Syn *in vivo*. Compared with α-Syn transgenic (Syn^Tg^) mice, TG2 null /α-Syn transgenic mice (TG2^KO^/Syn^Tg^) exhibited a reduced amount of phosphorylated α-Syn aggregates and fewer proteinase K-resistant α-Syn aggregates in sections of brain tissue. Neuritic processes that are depleted in Syn^Tg^ mice compared to wild-type mice were preserved in double TG2^KO^/Syn^Tg^ mice. Additionally, the neuroinflammatory reaction to α-Syn was attenuated in TG2^KO^/Syn^Tg^ animals. These neuropathological markers of diminished α-Syn toxicity in the absence of TG2 were associated with better motor performance on the rotarod and balance beam. These results suggest that deleting TG2 reduces the toxicity of α-Syn *in vivo* and improves the behavioral performance of Syn^Tg^ mice. Accordingly, these findings collectively support pharmacological inhibition of TG2 as a potential disease modifying therapeutic strategy for α-synucleinopathies.

## INTRODUCTION

Parkinson disease (PD) and Dementia with Lewy Bodies (DLB) are common neurodegenerative disorders characterized pathologically by intraneuronal aggregates of α-Synuclein (α-Syn) in Lewy bodies and Lewy neurites (Jenner and Olanow, 1998; Spillantini et al., 1998). α-Syn is a small, 140 amino acid intrinsically disordered protein (Lee and Trojanowski, 2006) that is prone to self-aggregate and forms fibrils in neuropathological hallmark inclusions in response to diverse exogenous and endogenous factors (Goldberg and Lansbury, 2000). This aggregation of α-Syn is believed to be a critical step leading to neuronal cell death (Goedert, 2001; Cookson, 2009). Thus, preventing α-Syn aggregation at an early stage is of therapeutic interest in α-synucleinopathies.

Transglutaminases (TGs) are a family of enzymes that catalyze a calcium-dependent formation of epsilon-(gamma-glutamyl) lysine isodipeptide bonds and result in a covalent linkage between two peptide molecules (Greenberg et al., 1991). Transglutaminase 2 (TG2) is one member of this family, which is expressed broadly in the mammalian and human brain in both neurons and astrocytes (Kim et al., 1999; Lesort et al., 1999). Several lines of evidence suggest that TG2 plays a pathogenic role in PD and DLB. In *in vitro* and cell culture studies, TG2 catalyzes the formation of high molecular weight α-Syn aggregates in a calcium dependent manner (Junn et al., 2003), and increased transamidation of α-Syn by TG2 is found in the 1-methyl-4-phenylpyridine (MPP(+)) toxicity model in SH-SY5Y cells (Verhaar et al., 2011; Grosso et al., 2014). In human studies, compared with control subjects, a significant increase in TG2 protein and mRNA expression is found in the substantia nigra of PD patients (Citron et al., 2002; Andringa et al., 2004; Wilhelmus et al., 2011) as well as increased TG2 protein levels in their cerebrospinal fluid (Vermes et al., 2004). Postmortem immunohistochemical and immunoblot studies have also shown the presence of isodipeptide bonds formed by TG2 co-localizing with α-Syn in Lewy bodies in both PD and DLB affected brains (Citron et al., 2002; Junn et al., 2003). Additionally, several pathogenic aberrations found in neurodegenerative disease brains, including oxidative stress, elevated calcium, and ATP depletion can activate TG2 (Grosso and Mouradian, 2012).

Based on this evidence, TG2 may impact the pathogenesis of PD and related disorders by contributing to α-Syn misfolding. However, whether TG2 depletion can mitigate the toxicity of α-Syn *in vivo* has not yet been demonstrated. In the present study, we show that genetic deletion of the TG2 gene in mice attenuates the accumulation of α-Syn aggregates, protects neurons from the toxicity of α-Syn overexpression, improves neuronal integrity, and reduces the associated neuroinflammation, leading to improved behavioral performance.

## EXPERIMENTAL PROCEDURES

### Animals

C57BL/6J mice were obtained from the Jackson Laboratories (Bar Harbor, ME). TG2 knockout mice (Nanda et al., 2001) and human wild-type α-synuclein transgenic mice under the control of the murine Thy-1 promoter (Rockenstein et al., 2002) are described previously. To create TG2^KO^/Syn^Tg^ double modified mice, α-Synuclein transgenic (Syn^Tg^) female mice were crossbred with male TG2^KO^ mice. The TG2^KO^ line was maintained by breeding TG2^KO^ mice with WT mice of the C57B/6 background. Genotypes were determined by PCR of tail DNA. Only male mice were used in this study. Mice were sacrificed, and brains were collected from 6 to 9 month old animals. All housing, breeding, and procedures were performed according to the NIH Guide for the Care and Use of Experimental Animals and approved by the Rutgers-Robert Wood Johnson Medical School Institutional Animal Care and Use Committee.

### Immunohistochemistry and immunofluorescence

Mice were perfused transcardially with PBS, and brains were removed and fixed in 10% formalin (Sigma-Aldrich) at 4 °C overnight. Brains were sectioned using a Leica VT1000 S vibratome at 40 μm thickness in the coronal plane through the entirety of the brain from the frontal association cortex through the pons, and serial sections were collected as sets with the same interval. Sections were then selected from this bank of tissue for each staining marker. For cortical and striatal studies, sections were selected at approximately Bregma 0.98 in the Paxinos and Franklin Mouse Brain Atlas using the anterior commissure and corpus callosum as landmarks to select equivalent sections across animals. For immunohistochemistry, free-floating sections were pretreated differently before adding antibodies depending on staining conditions. For regular immunohistochemistry, sections were blocked in 5% BSA (Sigma-Aldrich) following incubation with 3% hydrogen peroxide (Sigma-Aldrich) to inhibit endogenous peroxidase activity. For proteinase K treatment, samples were incubated in 88% formic acid (Thermo Fisher Scientific) for 10 min for antigen retrieval and then incubated in 10 μg/ml proteinase K (Sigma-Aldrich) for 10 min before being blocked in 5% BSA. After pretreatments, sections were incubated with α-synuclein antibody (#610787, BD bioscience) at 4 °C overnight and with biotinylated secondary antibody (Sigma-Aldrich) for 1 h at room temperature. Vectastain elite ABC kit (Vector Laboratories, Burlingame, CA, USA) and 3.3’-diaminobenzidine (Sigma-Aldrich) were used for amplification and color development. Images were captured using a Nikon Eclipse 55i microscope and NIS Elements D3.2 software (Nikon, Tokyo, Japan). For immunofluorescence staining of microtubule-associated protein 2 (MAP2), sections were blocked with 5% goat serum (Sigma-Aldrich) and 0.2 % Triton X-100 (Sigma-Aldrich) in PBS. Sections were then incubated with primary antibody overnight at 4 °C and fluorescent secondary antibody for 1 h at room temperature. Images were captured using Carl Zeiss Axiovert 200 microscope. For cortical studies, images were taken of the outer layers of the motor cortex. ImageJ was used to threshold stained areas and to automatically calculate the number of defined regions and total optical density (OD). Primary antibodies used were anti-α-synuclein (#610787, BD Bioscience), anti-phospho-Ser129-α-Syn (#015–25191, WAKO), anti-glial fibrillary acidic protein (GFAP) (Dako Carpinatria, CA, USA), anti-Ionized calcium-binding adaptor molecule 1 (Iba-1, a marker of microglial activation as an indication of neuroinflammation) (#019–19741, WAKO), and anti-microtubule-associated protein 2 (MAP2, a marker of neuritic processes as a reflection of neuronal integrity) (Santa Cruz Biotechnology).

### Behavioral assessments

Behavioral assessments were performed at 6 months of age. The rotarod test was done as described previously (Lee et al., 2015). Briefly, mice were placed on a rotating cylinder (diameter = 4.5 cm) with a coarse surface for firm grip and tested for 3 trials with an accelerating speed of 0.2 rpm/s, increasing from 4 to 40 rpm. A cutoff time of 3 min and an inter-trial interval of 60 min were used.

Latency on the rod before falling was measured. The balance beam test was as described before (Quinn et al., 2008). In brief, mice were habituated to a dark goal box for 3 min and then trained to walk across a narrow beam to reach that box. The following day, three consecutive trials were done, and the time taken to cross the beam to reach the goal box with each trial was recorded for each mouse.

### Statistical analysis

The results are presented as box-plots where the bottom and the top of the box are the first and third quartiles, respectively, and the whiskers above and below the box indicate the 95th and 5th percentiles. The median is indicated as a horizontal line. Data are analyzed by one-way factorial analysis of variance (ANOVA) followed by Tukey’s Multiple Comparison Test when comparing all four groups. Unpaired t test was used when only Syn^Tg^ and TG2^KO^/Syn^Tg^ mice were compared for p-α-Syn staining and proteinase K-resistant α-Syn staining. Significance was determined at *p* < 0.05. Statistical analyses were performed using GraphPad Prism 7.0 (GraphPad Software, Inc., San Diego, CA).

## RESULTS

### Genetic deletion of TG2 prevents the formation of α-Syn aggregates in the mouse brain

**Fig. 1.**
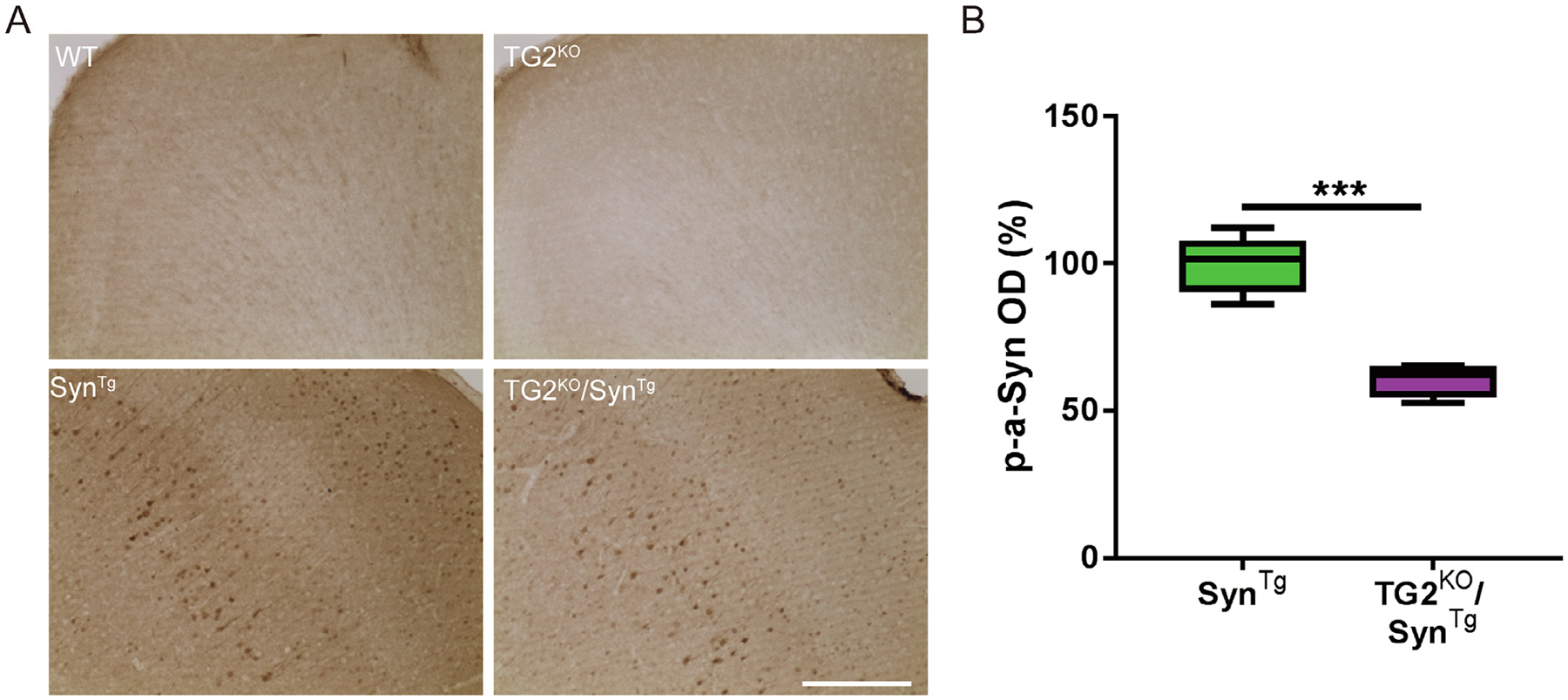
TG2 deletion decreases the formation of phosphorylated α-Syn in the mouse brain. **(A)** Representative images of p-α-Syn staining in the cortex of mice. **(B)** Quantification of p-α-Syn staining intensity in **(A)** (n: Syn^Tg^ = 6; TG2^KO^/Syn^Tg^ = 5). In the box-plots, the bottom and top of the box are the first and third quartiles, respectively, and the whiskers above and below the box indicate the 95th and 5th percentiles. The median is indicated as a horizontal line. ***P < 0.001, two-tailed unpaired t test. Scale bar = 50 μm.

Given that TG2 crosslinks α-Syn and promotes its aggregation (Junn et al., 2003; Grosso et al., 2014), we first assessed whether deletion of the TG2 gene impacts the formation of these aggregates in the brains of Syn^Tg^ mice using immunohistochemical stains. As α-Syn aggregates in both human α-synucleinopathies and in Syn^Tg^ mouse brains are characteristically hyperphosphorylated at serine 129 and are resistant to clearance by proteinase K (Neumann et al., 2002), antibody to phosphorylated α-Syn (p-α-Syn) and staining for α-Syn after digestion with proteinase K were employed to address this question. As expected, brain sections from TG2^KO^/Syn^Tg^ mice had lower phosphorylated α-Syn intensity in the cortex, by as much as half of that detected in Syn^Tg^ mice (Fig. 1A, B). Brains of wild-type and TG2^KO^ mice had no p-α-Syn immunoreactive neurons (Fig. 1A). Similarly, the number of proteinase K-resistant α-Syn aggregates in the striatum of TG2^KO^/Syn^Tg^ mice was 42% of that detected in Syn^Tg^ mice (Fig. S1A, B). No punctate aggregates were found in WT or TG2^KO^ mice following this protease digestion (Fig. S1A). These results suggest that deletion of TG2 prevents the formation of misfolded pathogenic species of α-Syn *in vivo*.

### TG2 depletion improves neuronal integrity that is lost in Syn^Tg^ mice

**Fig. 2.**
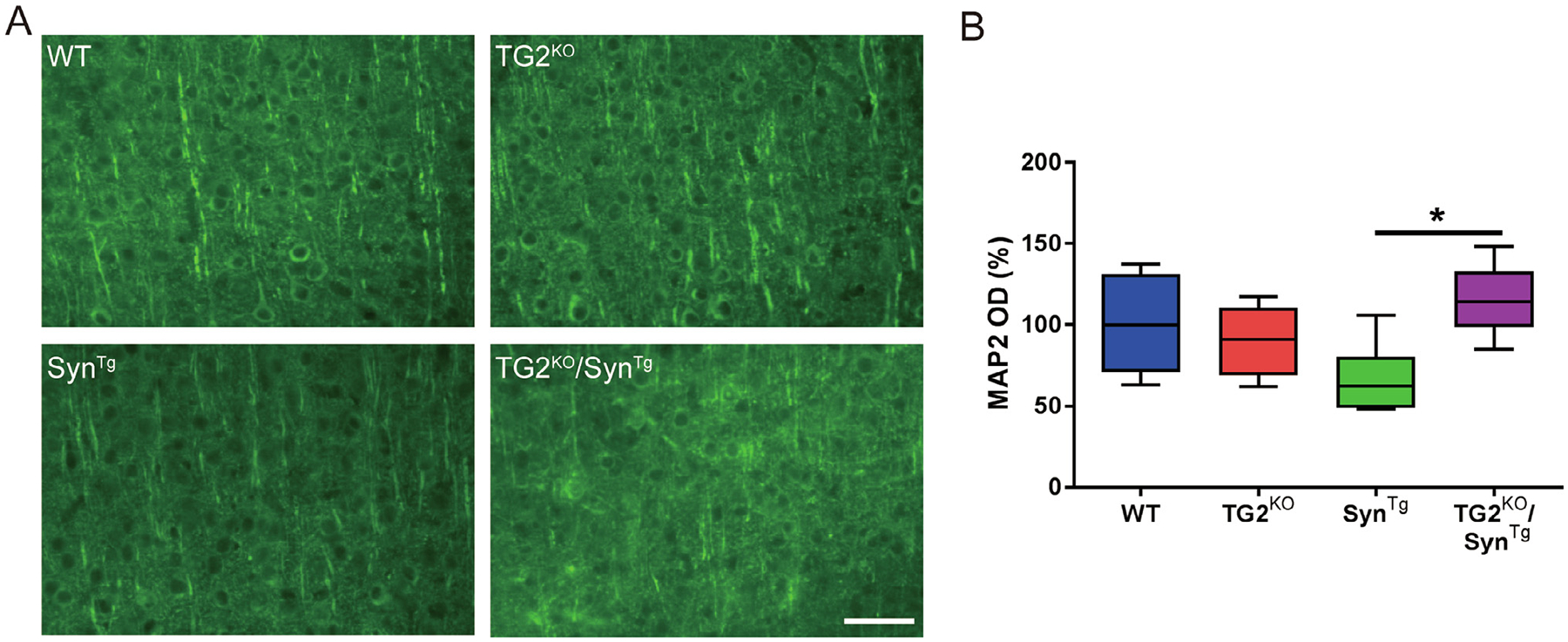
TG2 deletion protects against the neuronal toxicity of α-Syn. **(A)** Representative images of MAP2 staining in the cortex of mice. **(B)** Quantification of immunofluorescence staining of MAP2 in **(A)** (n: WT = 6; TG2^KO^ = 6; Syn^Tg^ = 6; TG2^KO^/Syn^Tg^ = 5). In the box-plots, the bottom and top of the box are the first and third quartiles, respectively, and the whiskers above and below the box indicate the 95th and 5th percentiles. The median is indicated as a horizontal line. *P < 0.05, one-way ANOVA. Scale bar = 50 μm.

We previously reported that TG2 exacerbates the neuronal toxicity of α-Syn *in vivo* (Grosso et al., 2014).To investigate whether TG2 deletion prevents the deleterious effect of α-Syn overexpression, MAP2 staining was assessed next. Syn^Tg^ mice have substantial depletion of MAP2 immunoreactivity in the cortex (Fig. 2A), suggestive of disrupted nerve fibers and reduced dendritic complexity (Harada et al., 2002; Koob et al., 2010; Lee et al., 2011). On the other hand, TG2 deletion prevented the degradation of neuritic complexity in Syn^Tg^ mice (Fig. 2A, B). These observations suggest that TG2 depletion protects against α-Syn induced neuronal toxicity.

### TG2 deletion attenuates the neuroinflammation in Syn^Tg^ mice

**Fig. 3.**
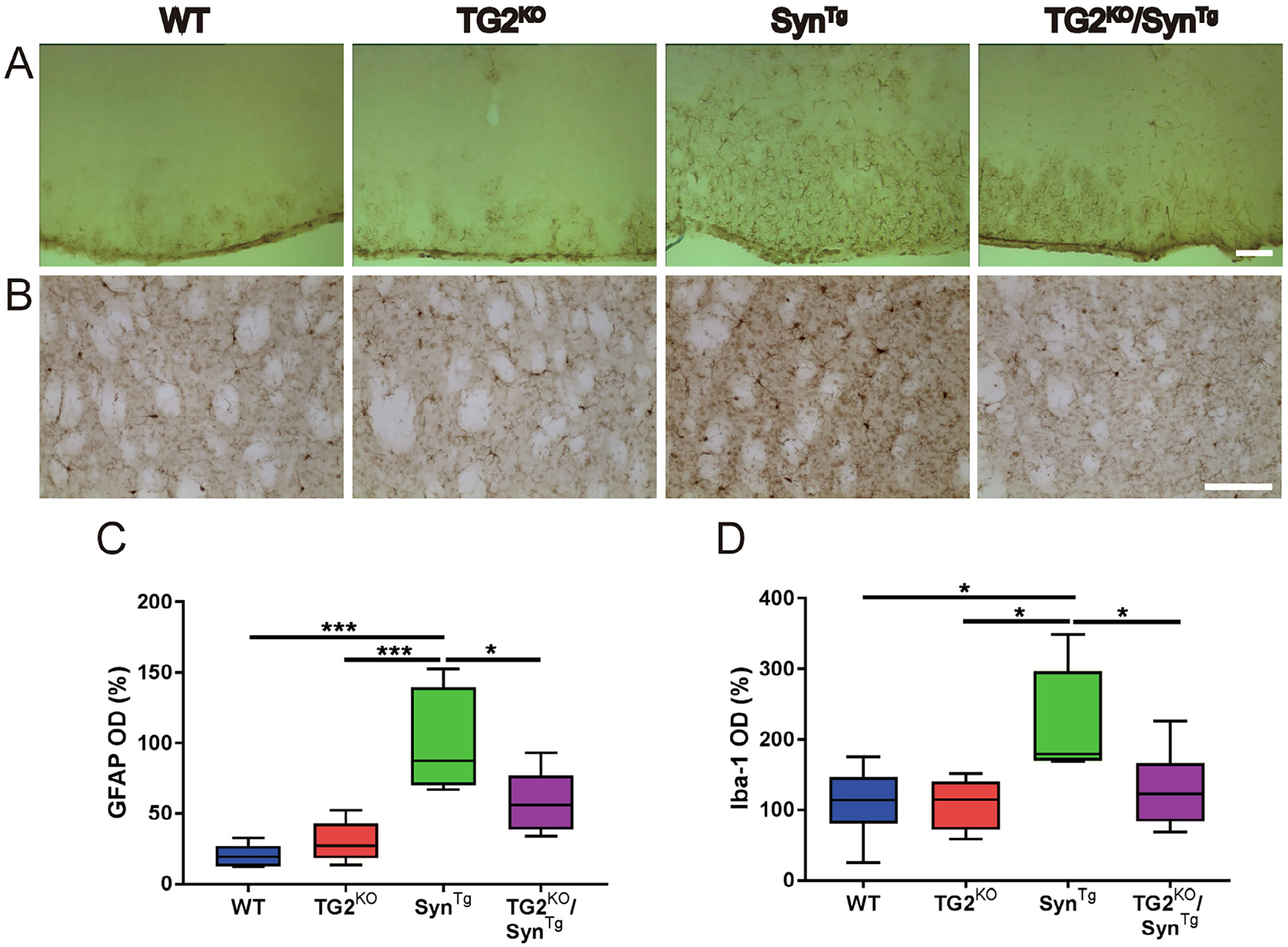
TG2 deletion prevents the neuroinflammatory response to α-Syn. **(A)** Representative immunohistochemical images of cortical sections from each of the four mouse lines stained for GFAP. **(B)** Representative images of striatal sections stained for Iba-1. **(C)** Quantification of immunohistochemical staining of GFAP in **(A)** (n: WT = 6; TG2^KO^ = 6; Syn^Tg^ = 6; TG2^KO^/Syn^Tg^ = 5). **(D)** Quantification of immunohistochemical staining of Iba-1 in **(B)** (n: WT = 6; TG2^KO^ = 6; Syn^Tg^ = 6; TG2^KO^/Syn^Tg^ = 6). In the box-plots, the bottom and top of the box are the first and third quartiles, respectively, and the whiskers above and below the box indicate the 95th and 5th percentiles. The median is indicated as a horizontal line. *P < 0.05; *** *P* < 0.001, one-way ANOVA. Scale bar = 50 μm.

Neuroinflammation is one of the neuropathological features of PD and DLB (Tansey and Goldberg, 2010; Surendranathan et al., 2018) as well as in models of α-synucleinopathy including Syn^Tg^ mice (Lee et al., 2011). We previously showed that TG2 aggravates the neuroinflammation in the brains of Syn^Tg^ mice (Grosso et al., 2014). To investigate whether deleting TG2 has the opposite effect, immunohistochemistry for the astrocytic marker GFAP (Fig. 3A) and the microglial marker Iba1 (Fig. 3B) were performed. Consistent with our previous findings (Lee et al., 2011; Grosso et al., 2014; Lee et al., 2015; Yan et al., 2018), Syn^Tg^ mice had a markedly increased GFAP positive signal in the cortex compared with WT and TG2^KO^ mice, while genetic deletion of TG2 significantly decreased GFAP signal induced by overexpression of α-Syn (Fig. 3A, C). Similarly, Syn^Tg^ mice had significantly increased staining of the microglial marker Iba1 compared to the other 3 genotypes including TG2^KO^/Syn^Tg^ mice (Fig. 3B, D). These findings suggest that TG2 deletion can mitigate the neuroinflammatory response to α-Syn.

### Deleting TG2 prevents the behavioral deficits of Syn^Tg^ mice

**Fig. 4.**
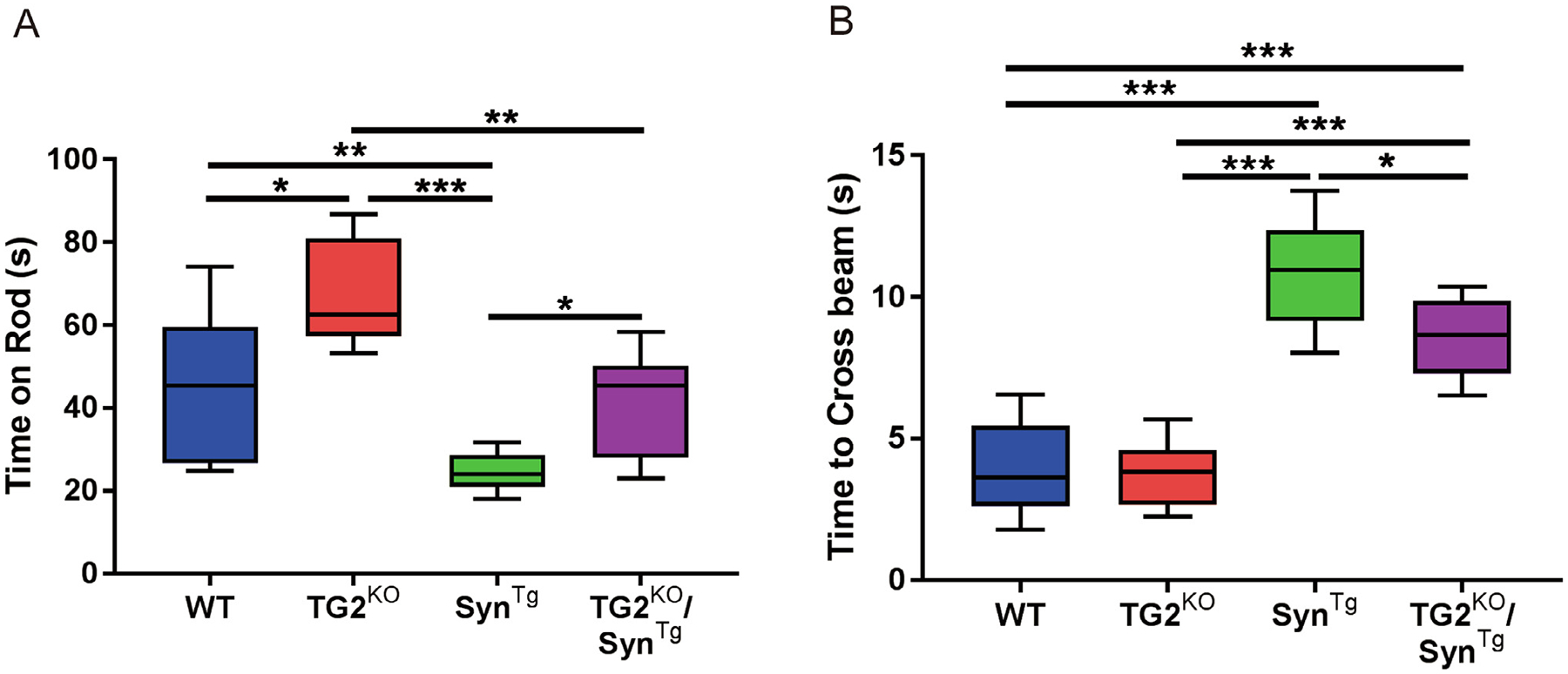
TG2 deletion prevents the behavioral deficits of Syn^Tg^ mice. **(A)** Performance on the rotarod (n: WT = 11; TG2^KO^ = 6; Syn^Tg^ = 9; TG2^KO^/Syn^Tg^ = 10). **(B)** Time taken to cross the balance beam (n: WT = 11; TG2^KO^ = 6; Syn^Tg^ = 9; TG2^KO^/Syn^Tg^ = 6). In the box-plots, the bottom and top of the box are the first and third quartiles, respectively, and the whiskers above and below the box indicate the 95th and 5th percentiles. The median is indicated as a horizontal line. *P < 0.05; **P < 0.01; ***P < 0.001, one-way ANOVA.

To determine if the neuropathological markers of protection associated with TG2 deletion correlate with improved motor behavior, performance on the balance beam test and the rotarod were evaluated at 6 months of age. Both tasks were chosen because they reflect nigrostriatal function (Rozas and Labandeira García, 1997; Quinn et al., 2008). As expected, Syn^Tg^ mice showed significantly impaired ability to stay on the rotarod compared to WT and TG2^KO^ mice, whereas the performance of double modified TG2^KO^/Syn^Tg^ mice was significantly better (Fig. 4A). A similar profile of differences in the balance beam test was found. Syn^Tg^ mice had worse performance than WT and TG2^KO^ mice, while TG2^KO^/Syn^Tg^ mice demonstrated less severe impairment compared to Syn^Tg^ mice (Fig. 4B). These behavioral improvements in TG2^KO^/Syn^Tg^ mice are consistent with the histopathologic data in these animals.

## DISCUSSION

The present findings demonstrate that deleting the TG2 gene in Syn^Tg^ mice results in reduced α-Syn aggregation in the brain, preserved neuronal integrity and less intense neuroinflammation, as well as better motor performance. These findings together suggest that deletion of TG2 attenuates the toxicity of α-Syn.

α-Syn plays a key role in both familial and sporadic forms of PD based on several lines of evidence from genetic, neuropathologic and cellular/molecular studies (Lee and Trojanowski, 2006; Bridi and Hirth, 2018). Due to its natively unfolded conformation, α-Syn tends to self-aggregate and accumulate in Lewy bodies and Lewy neurites (Paik et al., 1998; Goldberg and Lansbury, 2000). *In vitro* experiments have shown that α-Syn can form aggregates and assemble into elongated filaments (Giasson et al., 1999; Uversky et al., 2002; Yang et al., 2019), which is consistent with the high molecular weight (HMW) α-Syn found in α-Syn transgenic mice (Grosso et al., 2014; Lee et al., 2015). The fact that TG2 augments the accumulation of these HMW species has been demonstrated in both *in vitro* and *in vivo* studies (Junn et al., 2003; Grosso et al., 2014). The present study shows that deletion of TG2 prevents the formation of α-Syn aggregates in syn^Tg^ mice where the protein is overexpressed in neurons. This finding complements our previous observation that over-expression of TG2 enhances α-Syn aggregation in the mouse brain (Grosso et al., 2014), thus further supporting the conclusion that TG2 promotes α-Syn aggregation *in vivo*.

The pathology of PD is associated with a neuroinflammatory reaction, and α-Syn-induced neuroinflammation is well documented. Microglia are activated with elevated inflammatory cytokines in Syn^Tg^ animals under the control of the pan-neuronal Thy-1 promoter (Grosso et al., 2014; Lee et al., 2015) or the dopamine neuron specific tyrosine hydroxylase promoter (Richfield et al., 2002). Localized overexpression of α-Syn using Adeno-Associated Virus vector-mediated delivery also activates microglia (Sanchez-Guajardo et al., 2010). In addition to microglial activation, overexpression of α-Syn in mice induces astrocytic activation (Lee et al., 2011; Kurz et al., 2012). We previously demonstrated that TG2 overexpression aggravates the neuroinflammatory effects of α-Syn *in vivo*, including activation of microglia and astrocytes (Grosso et al., 2014). Here, we demonstrate that deleting TG2 attenuates the neuroinflammation in Syn^Tg^ mice, which strongly supports the involvement of TG2 in α-Syn induced neuroinflammation.

Impaired neuronal function and morphology in Syn^Tg^ mice indicate α-Syn toxicity, which is detected by depletion of MAP2 and impaired motor performance (Harada et al., 2002; Koob et al., 2010; Lee et al., 2011). Exacerbation of α-Syn-mediated neuronal toxicity due to TG2 over-expression was demonstrated in our previous study (Grosso et al., 2014). The reciprocal finding in the present study shows that depletion of TG2 prevents the neurotoxicity of α-Syn by maintaining neuronal integrity and minimizing the motor behavioral deficits of Syn^Tg^ mice. Thus, the present results confirm that TG2 impacts the toxicity of α-Syn *in vivo*.

In conclusion, our data provide evidence that deleting TG2 mitigates the toxicity of α-Syn and its downstream neuropathologic consequences. These findings extend and support our previous study that shows TG2 exacerbates α-Syn toxicity in mice and yeast (Grosso et al., 2014). Thus, the experimental evidence from two studies collectively suggests that inhibiting TG2 is a plausible disease modifying therapeutic strategy for PD and related α-synucleinopathies.

## Supplementary Material

1

## Abbreviations:

ANOVA: analysis of variance
DLB: dementia with lewy bodies
GFAP: glial fibrillary acidic protein
HMW: high molecular weight
Iba-1: ionized calcium-binding adaptor molecule 1
MAP2: microtubule-associated protein 2
MPP(+): 1-methyl-4-phenylpyridine
PD: Parkinson disease
p-α-Syn: phosphorylated α-Syn
Syn^Tg^: α-Syn transgenic
TG2: transglutaminase 2
TG2^KO^: TG2 null
α-Syn: α-Synuclein
