# Supplementary figures and images for: Transglutaminase 2 Depletion Attenuates α-Synuclein Mediated Toxicity in Mice
Source: Neuroscience. Author manuscript; Available in PMC 2021 Apr 6. (PMC8024061; doi:10.1016/j.neuroscience.2020.05.047)

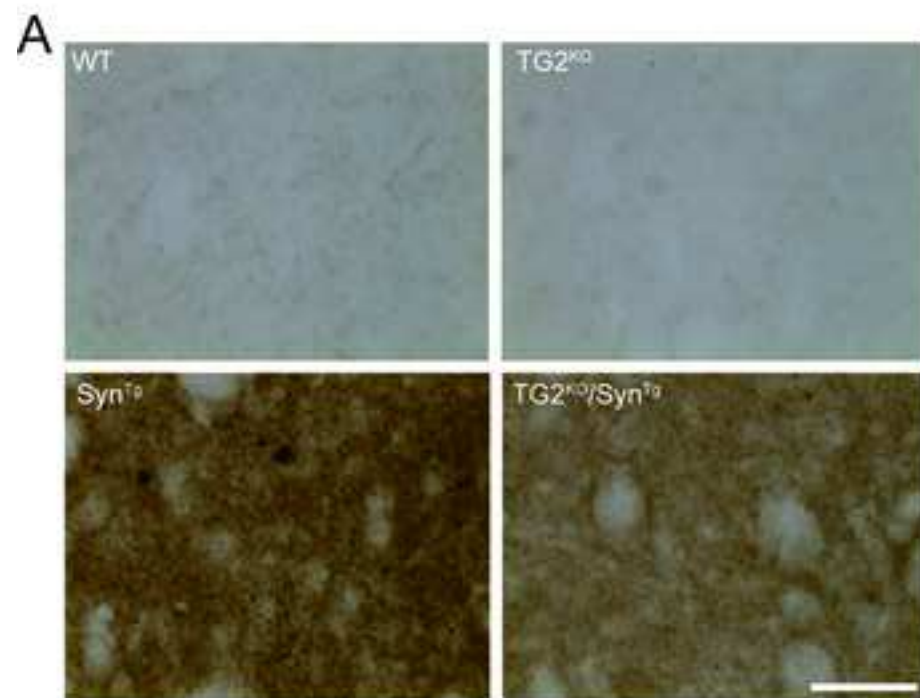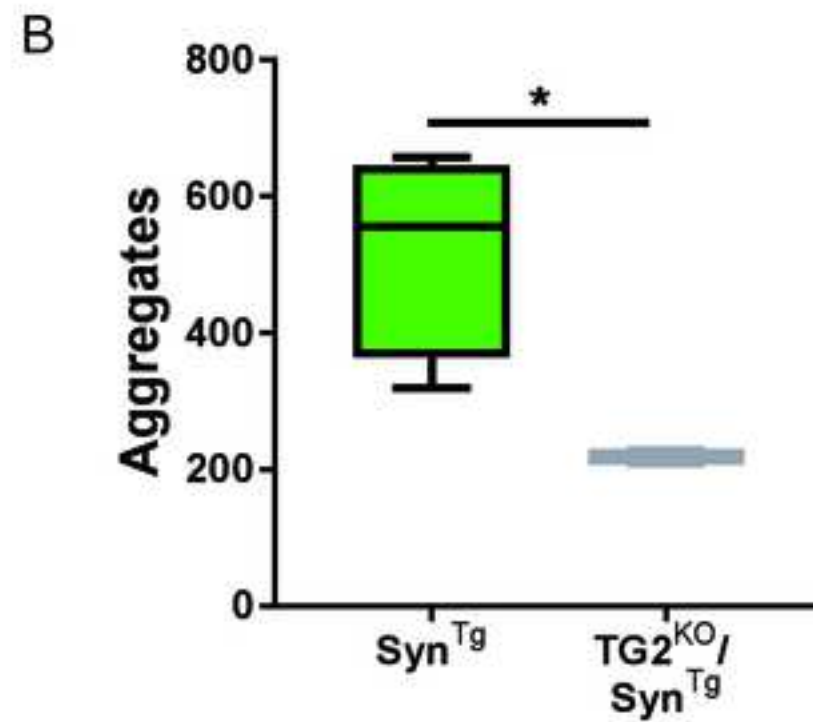

Supplement: 1 [file NIHMS1683917-supplement-1.pdf]
